# Antifungal active ingredient from the twigs and leaves of *Clausena lansium* Lour. Skeels (Rutaceae)

**DOI:** 10.3389/fchem.2022.1104805

**Published:** 2022-12-13

**Authors:** Xiaoxiang Fu, Suling Xiao, Duantao Cao, Minxuan Yuan, Miaolian Xiang, Qinghong Zhou, Yingjin Huang, Hongyi Wei, Wenwen Peng

**Affiliations:** ^1^ The Laboratory for Phytochemistry and Botanical Pesticides, College of Agriculture, Jiangxi Agricultural University, Nanchang, China; ^2^ Jiangxi Province Key Laboratory of Tuberous Plant Biology, Jiangxi Agricultural University, Nanchang, China; ^3^ Key Laboratory of Crop Physiology, Ecology and Genetic Breeding, Ministry of Education/Jiangxi Province, Jiangxi Agricultural University, Nanchang, China

**Keywords:** Clausena lansium, amide, coumarin, antifungal activity, structure-activity relationships

## Abstract

Two novel amides, named clauphenamides A and B, and twelve other known compounds were isolated from the twigs and leaves of *Clausena lansium* Lour. Skeels (Rutaceae). Their structures were elucidated on the basis of extensive spectroscopic analysis and comparison with data reported in the literature. Clauphenamide A (**1**) featured in the unit of N-2-(4,8-dimethoxyfuro [2,3-b]quinolin-7-yl)vinyl, and clauphenamide B (**2**) was a unprecedented N-phenethyl cinnamide dimer. Other known compounds belong to pyrrolidone amides (**3** and **4**), furacoumarins (**7**–**10**), simple coumarins (**11**–**14**), lignan (**5**) and sesquiterpene (**6**). Compounds **5**, **6**, **10** and **12** were separated from the genus (*Clausena*) for the first time, while **13** was isolated in the species (*C. lansium*) for the first time. The antifungal activities of the isolated compounds were assayed. As a result, at the concentration of 100 *μ*g/ml, compared with the control (chlorothalonil, inhibition rate of 83.67%), compounds **1** and **2** were found to exhibit moderate antifungal activity against *B. dothidea* with inhibition rates of 68.39% and 52.05%, respectively. Compounds **11**–**14** also exhibited moderate activity against *B. dothidea* and *F. oxysporum*, with inhibition rates greater than 40%. In addition, compared with the control (chlorothalonil, inhibition rate of 69.02%), compounds **11**–**14** showed strong antifungal activity to *P. oryzae*, with inhibition rates greater than 55%. Among them, compound **14** has the strongest antifungal activity against *P. oryzae*, and the inhibition rate (65.44%) is close to that of the control chlorothalonil. Additionally, the structure-activity relationships of the separated compounds are also discussed preliminarily in this paper.

## Introduction

*Clausena lansium* Lour. Skeels (Rutaceae), native to southern China and now distributed throughout the subtropical and tropical regions, is one of approximately 30 members of the genus *Clausena* (Rutaceae) (Editorial Committee, 1997; Peng et al., 2019). This plant is famous for its good medicinal value and delicious fruit. Previous phytochemical investigation on *C. lansium* has revealed that the chemical constituents of *C. lansium* are diverse, including alkaloids (Peng et al., 2018), coumarins (Peng et al., 2021), amides (Peng et al., 2020), sesquiterpenes (Liu et al., 2021), sesquiterpene glycosides (Peng et al., 2019), aromatic glycosides (Peng et al., 2019) and so on, endowing diverse pharmacological activities such as the antitumor, antifungal, antioxidant, hypoglycemic, nematicidal, hepatoprotectiv, neuroprotective, antiobesity, antimicrobial, and anti-inflammatory for this plant (Shen et al., 2012; Deng et al., 2014a; 2014b; Liu et al., 2014; Shen et al., 2014; Song et al., 2014; Xu et al., 2014; Du et al., 2015; Huang et al., 2017; Fan et al., 2018; Yan et al., 2018).

Amides are important active components in *C. lansium*, which are divided into cyclic amides (Yang et al., 1988), phenylpropionamides (Lin, 1989; Milner et al., 1996) and other amides (Peng et al., 2020). More than twenty amides have been isolated from *C. lansium* since 1988 (Yang et al., 1988; Liu et al., 1996; Lin, 1989; Milner et al., 1996), which exhibit a variety of biological activities, such as hepatoprotective, hypolipidemia, antispasmodic (Moshi et al., 2005), anti HIV (Sunthitikawinsakul et al., 2003), immunomodulatory (Manosroi et al., 2005), antiviral (Adebajo et al., 2009), antimalaria (Okokon et al., 2012), and cytotoxic (Sripisut et al., 2012) activities. Besides, amides from *C. lansium* also show the potential for development and utilization in pesticide activities, including insecticidal (Cheng et al., 2010; Han et al., 2013; Ramkumar et al., 2015), antifungal (Ng et al., 2003; Li et al., 2014; Yan et al., 2018) and phytocidal (Peng et al., 2021) effects.

Coumarins are another important active ingredient in *C. lansium*, mainly furacoumarins (Ito et al., 1998; Peng et al., 2021), which exhibit hypoglycemic (Zhang et al., 2012), anti-tumor (Prasad et al., 2010), antibacterial (Tada et al., 2002), herbicidal (Peng et al., 2021) and other activities.

**FIGURE 1:**
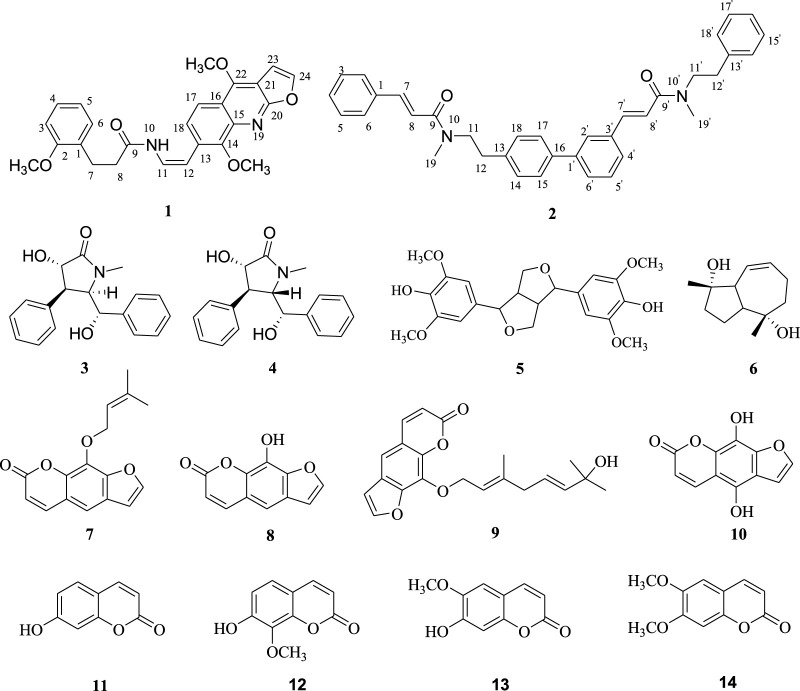
Structures of amides compounds **1**–**14**

In the early stage, we carried out a detailed investigation on the chemical substances of *C. lansium*, and isolated various chemical components, including alkaloids (Peng et al., 2018), coumarins (Peng et al., 2021), sesquiterpenes (Liu et al., 2021), sesquiterpene glycosides (Peng et al., 2019), aromatic glycosides (Peng et al., 2019), amides (Song et al., 2014; Peng et al., 2020) and so on. As part of our continuous efforts to find new bioactive natural products, especially amides, alkaloids and coumarins, from *C. lansium*, a continuing chemical investigation on the twigs and leaves of *C. lansium* was carried out in the current work, leading to the isolation of four amides (**1**–**4**), eight coumarins (**7**–**14**), one lignan (**5**) and one sesquiterpene (**6**) (Figure 1). Compoud **1** was one unique amide with the unit of N-2-(4,8-dimethoxyfuro [2,3-b]quinolin-7-yl)vinyl, and **2** was a unprecedented N-phenethyl cinnamide dimer. Compounds **5**, **6**, **10** and **12** were separated from *Clausena* for the first time, while **13** was isolated in *C. lansium* for the first time. All compounds were evaluated for their antifungal activities against *Botryosphaeria dothidea* (Moug.) Ces. and De Not., *Fusarium oxysporum* and *Pyricularia oryzae* Cav. *via* a mycelial growth inhibition assay. In this paper, we described the isolation, identification, and antifungal activities screening and structure-activity relationships of the above mentioned chemical composition from *C. lansium*.

## Materials and methods

### General experimental procedures

UV spectra were obtained using a polarimeter (Horiba SEPA-300) (Horiba, Tokyo, Japan). An FT-IR spectrometer (Tensor 27 with KBr pellets) (BioRad, Hercules, CA, United States) was used to record IR spectra of compounds. NMR spectra were recorded with an instrument (Bruker Avance AV-400) (Switzerland, Bruker A.G.) at room temperature. HRESIMS data were obtained with a spectrometer (a Bruker Daltonics Inc. micro-TOF-Q). Reverse-phase medium-pressure liquid chromatography (RP-MPLC) was performed on a Buchi RP-MPLC instrument (Buchi Labortechnik AG, Flawil, Switzerland) with a YMC gel ODS column (50 *μ*m, YMC Co., Ltd., Kyoto, Japan). Semipreparative high-performance Liquid Chromatography (HPLC) was performed on an Agilent 1260 instrument (Agilent, Palo Alto, CA, United States) with a UV detection and a column (Agilent Eclipse, XDB-C18, 5 μm, 9.4 × 250 mm). Column chromatography (CC) was carried out on silica gel (100–200 mesh, 200–300 mesh) (Qingdao Marine Chemical, Inc., Qingdao, China) and Sephadex LH-20 (GE Healthcare Bio-Sciences AB, Uppsala, Sweden). TLC was performed with glass-precoated silica gel GF_254_ plates (Qingdao Marine Chemical Factory, China). The organic solvents were purchased from Sinopharm Chemical Reagent Co., Ltd. (Shanghai, China).

### Plant material

The twigs and leaves of *C. lansium* were collected from Qingyuan county (23°70′ N, 113°03′ E), Guangdong Province, China, in October 2015, and identified by Prof. Zhou Xin-xin, South China Botanical Garden, Chiese Academy of Sciences, Guangdong, China. A voucher specimen (no. 2015912) has been deposited in the Laboratory for Phytochemistry and Plant-derived Pesticides, College of Agriculture, Jiangxi Agri-cultural University.

### Extraction and isolation

The air-dried twigs and leaves of *C. lansium* (11 kg) were crushed into a powder and extracted with refluxing 95% methanol (3 × 20 L, 6 h each time). The methanol extract was suspended in water (5 L) and partitioned with petroleum ether (PE), ethyl acetate (EtOAc) and n-butyl alcohol (n-BuOH) (3 × 5 L, each) to afford PE extract (210 g), EtOAc extract (890 g), and n-BuOH extract (130 g), respectively. The EtOAc part was chromatographed on a silica gel column (100–200 mesh) and eluted with a gradient mixture of PE-Acetone (1: 0 to 0: 1) to provide six major fractions: (Fr. A-F).

Fr. D (16.4 g) was subjected to RP-MPLC (MeOH/H_2_O, 20–100%) to give 9 fractions (Fr.D1−Fr.D9). Fr. D2 (57.2 mg) was chromatographed on silica gel column (200–300 mesh) eluted with a isocratic system of PE–Acetone (4:1) to give a mixture (33.1 mg) of compounds **3** and **4**. The mixture was isolated and purified by HPLC (MeOH–H_2_O 73:27) to yield **3** (6 mg, tR = 23.3 min) and **4** (7 mg, tR = 26.5 min). Fr. D3 (43.1 mg) was further fractionated by Sephadex LH-20 column chromatography with MeOH and CH_2_Cl_2_ (1:1) to obtain three subfractions (Fr.D3.1 to Fr. D3.3), compounds **1** (5 mg, tR = 20.6 min) and **2** (6 mg, tR = 23.2 min) were obtained from Fr. D3.2 and Fr. D3.3 by Semipreparative HPLC (MeOH–H_2_O 75:25 and 76:24), respectively. Fr. D4 (57.8 mg) was chromatographed on silica gel column (200–300 mesh) eluted with a gradient system of PE–Acetone (5:1–3:1) to give five subfractions (Fr.D4.1 to Fr. D4.5). Then Fr. D4.2-Fr.D4.4 were repeatedly purified by Semipreparative HPLC (C₂H₃N-H_2_O 65:35–75:25). Finally, **8** (7 mg, C₂H₃N- H_2_O 60:40, tR = 21.2 min), **10** (6 mg, C₂H₃N-H_2_O 70:30, tR = 25.4 min) and **14** (5 mg, C₂H₃N-H_2_O 72:28, tR = 20.3 min) were obtained from Fr. D4.2, Fr. D4.3 and Fr. D4.4, respectively.

Fr. E (21.3 g) was subjected to RP-MPLC (MeOH/H_2_O, 30–100%) to give 6 fractions (Fr.E1−Fr.E6). Fr. E2 (91 mg) was further fractionated on silica gel column (200–300 mesh) eluted with a gradient system of PE–Acetone (5:1–3:1) to yield four subfractions (Fr.E2.1 to Fr. D2.4). Compounds **11** (6 mg, tR = 23.2 min) and **13** (9 mg, tR = 24.5 min) were isolated from Fr. E2.2 and Fr. E2.3 by Semipreparative HPLC (C₂H₃N–H_2_O 65:35 and 70:30), respectively. Similarly, Fr. E3 (82 mg) was subjected to silica gel column (200–300 mesh) eluted with a gradient system of PE–Acetone (5:1–3:1) to give three subfractions (Fr.E3.1 to Fr. D3.3). Fr. E3.2 was purified by repeated column chromatography (200–300 mesh, PE–Acetone 4:1–3:1) and Semipreparative HPLC (C₂H₃N–H_2_O 68:32) to obtain compound **5** (8 mg, tR = 23.6 min). Fr. E4 (75 mg) was separated by column chromatography (PE–Acetone 4:1–3:1) to give four subfractions (Fr.E4.1 to Fr. D4.4), then Fr. D4.2 was purified by Semipreparative HPLC (C₂H₃N–H_2_O 64:36) to give **12** (5 mg, tR = 21.3 min).

Fr. F (9.7 g) was subjected to RP-MPLC (MeOH/H_2_O, 30–100%) to give five fractions (Fr.F1−Fr.F5). Fr.F2 (39 mg) was subjected to silica gel column (200–300 mesh) eluted with a Isocratic system of PE–Acetone (4:1), and then was purified by Semipreparative HPLC (C₂H₃N–H_2_O 72:28) to give **6** (7 mg, tR = 18.7 min). Fr.F3 (110 mg) and Fr.F4 (99 mg) were repeated chromatographed on silica gel column (200–300 mesh) eluted with a isocratic system of PE–Acetone (4:1) to give **7** (34 mg) and **9** (14 mg), respectively.

### Spectroscopic data

Clauphenamide A (**1**): yellowish needles; UV (MeOH): *λ*max nm: 221, 266, 304; IR*ν*_max_ 3243, 2925, 1641, 1615, 1576, 1493 cm^−1^; ^1^H and ^13^C NMR spectroscopic data see Table 1; positive ion HRESIMS *m/z* 455.1584 [M + Na]^+^ (calcd. For C_25_H_24_N_2_O_5_Na, 455.1582).

**TABLE 1:** ^1^H (400 MHz) and ^13^C (100 MHz) NMR Data of. **1** and **2** in CDCl_3_ (*δ*, ppm, *J*/Hz).

Position	1		2	
	*δ*_H_ (*J* in Hz)	*δ*_C_	δ_H_ (*J* in Hz)	δ_C_
1		134.7 (s)		135.3 (s)
2		158.4 (s)	7.05–7.38 (1H, m)	126.9 (d)
3	6.76 (d, 8.1)	114.2 (d)	7.05–7.38 (1H, m)	128.9 (d)
4	7.38 (t, 8.1)	131.4 (d)	7.05–7.38 (1H, m)	128.6 (d)
5	7.34 (overlapped)	129.8 (d)	7.05–7.38 (1H, m)	128.9 (d)
6	6.96 (d, 7.7)	107.8 (d)	7.05–7.38 (1H, m)	126.9 (d)
7	2.77 (t, 6.5)	34.8 (t)	7.61 (d, 15.5)	142.4 (d)
8	3.58 (t, 6.5)	41.3 (t)	6.76 (d, 15.5)	117.6 (d)
9		167.5 (s)		166.9 (s)
10	6.08 (br s)			
11	7.05 (d, 8.3)	128.6 (d)	3.60 (2H, t, 7.3)	51.9 (t)
12	7.59 (d, 8.3)	126.8 (d)	2.82 (2H, t, 7.3)	36.3 (t)
13		130.9 (s)		138.1 (s)
14		156.9 (s)	7.05–7.38 (1H, m)	128.6 (d)
15		137.6 (s)	7.05–7.38 (1H, m)	127.8 (d)
16		119.7 (s)		129.6 (s)
17	7.75 (d, 8.6)	114.2 (d)	7.05–7.38 (1H, m)	127.8 (d)
18	7.25 (d, 8.6)	123.5 (d)	7.05–7.38 (1H, m)	128.6 (d)
19			2.95 (s)	34.7 (q)
20		163.3 (s)		
21		103.9 (s)		
22		154.6 (s)		
23	6.98 (d, 2.3)	104.6 (d)		
24	7.54 (d, 2.3)	143.9 (d)		
2-OCH_3_	3.98 (s)	56.0 (q)		
14-OCH_3_	3.69 (s)	55.3 (q)		
22-OCH_3_-	4.35 (s)	59.0 (q)		
1′				129.6 (s)
2′			7.05–7.38 (1H, m)	127.6 (d)
3′				135.3 (s)
4′			7.05–7.38 (1H, m)	126.2 (d)
5′			7.05–7.38 (1H, m)	129.4 (d)
6′			7.05–7.38 (1H, m)	127.6 (d)
7′			7.45 (d, 15.5)	141.8 (d)
8′			6.46 (d, 15.5)	117.3 (d)
9′				166.7 (s)
11′			3.60 (2H, t, 7.3)	50.3 (t)
12′			2.82 (2H, t, 7.3)	35.2 (t)
13′				139.2 (s)
14′			7.05–7.38 (1H, m)	127.8 (d)
15′			7.05–7.38 (1H, m)	128.9 (d)
16′			7.05–7.38 (1H, m)	126.9 (d)
17′			7.05–7.38 (1H, m)	128.9 (d)
18′			7.05–7.38 (1H, m)	127.8 (d)
19′			2.95 (s)	33.6 (q)

Clauphenamide B (**2**): yellowish plates; UV (MeOH): *λ*_max_ nm: 216, 221, 280; IR*ν*_max_ 2911, 1637, 1605, 1572, 1491 cm^−1^; ^1^H and ^13^C NMR spectroscopic data see Table 1; positive ion HRESIMS *m/z* 551.2676 [M + Na]^+^ (calcd. For C_36_H_36_N_2_O_2_Na, 551.2675).

Compound **3**: white solid, ESI-MS (positive ion) m/z 617 [2M + Na]^+^. ^1^H NMR (400 MHz, pyridine-d_5_) *δ*_H_ 7.47–7.02 (m, 10H, aromatic H), 5.23 (d, *J* = 2.3 Hz, 1H, H-7), 4.83 (d, *J* = 10.7 Hz, 1H, H-3), 4.55 (dd, *J* = 8.6, 2.3 Hz, 1H, H-5), 4.05 (dd, J = 10.7, 8.6 Hz, 1H, H-4), 3.34 (s, 3H, H-6); ^13^C NMR (100 MHz, pyridine-d_5_) *δ*_C_ 175.8 (s, C-2), 142.4 (s, C-1″), 137.4 (s, C-1′), 129.7 (d, C-3′, 5′), 128.6 (d, C-3″, 5″), 128.2(d, C-2′, 6′), 127.8 (d, C-2″, 6″), 127.5 (d, C-4′), 127.0 (d, C-4″), 73.2 (d, C-7), 70.4 (d,C-3), 66.7 (d, C-5), 51.3 (d, C-4), 31.1 (q, C-6).

Compound **4**: white solid, ESI-MS (positive ion) *m/z* 320 [M + Na]^+^, 617 [2M + Na]^+^. ^1^H NMR (400 MHz, MeOD) *δ*_H_ 7.30 (d, *J* = 7.4 Hz, 2H, H-aromatic), 7.13 (t, *J* = 7.4 Hz, 2H, H- aromatic), 7.04 (m, 4H, H-aromatic), 6.79 (d, *J* = 7.4 Hz, 2H, H-aromatic), 5.21 (d, *J* = 2.1 Hz, 1H, H-7), 4.08 (d, *J* = 6.0 Hz, 1H, H-3), 3.97 (m, 1H, H-5), 3.21 (t, *J* = 6.0 Hz, 1H, H-4), 3.04 (s, 3H, H-6). ^13^CNMR (100 MHz, MeOD) *δ*_C_ 175.8 (s, C-2), 142.7 (s, C-1″), 141.4 (s, C-1′), 129.3 (d,C-aromatic), 129.1 (d, C-aromatic), 128.3 (d, C-aromatic), 127.3 (d, C-aromatic),127.1 (d, C-aromatic), 78.9 (d, C-7), 70.9 (d, C-3), 70.1 (d, C-5), 48.2 (d, C-4), 28.7(q, C-6).

Compound **5**: colorless oil, ESI-MS (negative ion) *m/z* 418 [M]^−^. ^1^H NMR (400 MHz, CD_3_OD) *δ*_H_ 6.51 (4H, s, H-2, 2′, 6, 6′), 4.54 (2H, d, J = 3.9 Hz, H-7, 7′), 4.10 (2H, m, Ha-9, 9′), 3.72 (2H, dd, *J* = 9.2, 2.8 Hz, Hb-9, 9′), 3.67 (12H, s, H-OCH_3_), 2.97 (2H, br. s, H-8, 8′). ^13^C NMR (100 MHz, CD_3_OD) *δ*_C_ 149.2 (s, C-3, 3′, 5, 5′), 135.8 (s, C-4, 4′), 133.1 (s, C-1, 1′), 104.3 (d, C-2, 2′, 6, 6′), 87.5 (d, C-7, 7′), 72.6 (t, C-9, 9′), 56.6 (q, C-OCH3), 55.3 (d,C-8, 8′).

Compound **6**: white powder. ESI-MS (positive ion) *m/z* 219 [M + Na]^+^. ^1^H NMR (400 MHz, CDCl_3_) *δ*_H_ 5.76 (dt, *J* = 7.2, 5.4 Hz, 1H, H-8), 5.71 (d, *J* = 11.3 Hz, 1H, H-9), 2.24 (m, 2H, H-7a, 10), 2.00 (m, 2H, H-7b, 4), 1.69 (m, 6H, H-2, 3, 6), 1.23 (s, 3H, H-12), 1.16 (s, 3H, H-11). ^13^C NMR (100 MHz, CDCl_3_) *δ*_C_ 131.6 (d, C-8), 130.3 (d, C-9), 80.1 (s, C-1), 75.1 (s, C-5), 51.2 (d, C-10), 50.4 (d, C-4), 42.5 (t, C-6), 40.2 (t, C-2), 23.6 (t, C-7), 22.5 (q, C-11), 21.7 (t, C-3), 21.6 (q, C-12).

Compound **7**: yellow solid. ESI-MS (positive ion) *m/z* 293 [M + Na]^+^. ^1^H NMR (400 MHz, pyridine-d_5_) *δ*_H_ 7.97 (d, *J* = 2.1 Hz, 1H, H-2′), 7.86 (d, *J* = 9.6, 1H, H-4), 7.38 (s, 1H, H-5), 6.90 (d, *J* = 2.1 Hz, 1H, H-3′), 6.46 (d, J = 9.6 Hz, 1H, H-3), 5.61 (t, *J* = 7.1 Hz, 1H, H-2″), 5.09 (d, J = 7.1 Hz, 2H, H-1″), 1.65 (s, 6H, H-4″, 5″). ^13^C NMR (100 MHz, pyridine-d_5_) *δ*_C_ 160.5 (s, C-2), 148.6 (d, C-7), 147.6 (d, C-2′), 145.1 (d, C-4), 144.2 (s, C-8a), 139.2 (s, C-3″), 131.9 (s, C-8), 126.4 (s, C-6), 120.6 (d, C-2″), 117.1 (s, C-4a), 114.7 (d, C-3), 114.3 (d, C-5), 107.3 (d, C-3′), 70.4 (t, C-1″), 25.7 (q, C-4″), 18.2 (q, C-5″).

Compound **8**: yellow solid, ESI-MS (positive ion) *m/z* 225 [M+ Na]^+^, 427 [2M + Na]^+^. ^1^H NMR (400 MHz, pyridine-d_5_) *δ*_H_ 8.00(d, *J* = 1.7 Hz, 1H, H-2′), 7.82 (d, *J* = 9.6 Hz, 1H, H-4), 7.23 (s, 1H, H-5), 6.90 (d, *J* = 1.7 Hz, 1H, H-3′), 6.44 (d, *J* = 9.6 Hz, 1H, H-3). ^13^C NMR (100 MHz, pyridine-d_5_) *δ*_C_ 161.2 (s, C-2), 147.4 (d, C-2′), 147.1 (s, C-7), 145.6 (d, C-4), 141.1 (s, C-8a), 132.6 (s, C-8), 126.2 (s, C-6), 117.2 (s, C-4a), 114.7 (d, C-3), 110.1 (d, C-5), 107.7 (d, C-3′).

Compound **9**: yellow solid. ESI-MS (positive ion) *m/z* 377 [M + Na]^+^. ^1^H NMR (400 MHz, CDCl_3_) *δ*_H_ 7.73 (d, *J* = 9.7 Hz, 1H, H-4), 7.64 (d, *J* = 2.0 Hz, 1H, H-2′), 7.32 (s, 1H, H-5), 6.77 (d, J = 2.0 Hz, 1H, H-3′), 6.29 (d, *J* = 9.7 Hz, 1H, H-3), 5.49 (m, 3H, H-2″, 5″, 6″), 4.93 (d, *J* = 7.0 Hz, 2H, H-1″), 2.60 (d, *J* = 6.6 Hz, 2H, H-4″), 1.58 (s, 3H, H-8″), 1.23 (s, 6H, H-9″, 10″). ^13^C NMR (100 MHz, CDCl_3_) *δ*_C_ 160.5 (s, C-2), 148.5 (s, C-7), 146.6 (d, C-2′), 144.4 (d, C-4), 143.9 (s, C-8a), 141.8 (s, C-3″), 140.3 (d, C-6″), 131.4 (s, C-8), 125.7 (s, C-6), 123.7 (d, C-5″), 120.2 (d, C-2″), 116.5 (s, C-4a), 114.5 (d, C-3), 113.3 (d, C-5), 106.6 (d, C-3′), 70.6 (s, C-7″), 70.1 (t, C-1″), 42.0 (t, C-4″), 29.5 (q, C-9″, 10″), 16.6 (q, C-8″).

Compound **10**: yellow solid. ESI-MS (positive ion) *m/z* 241 [M + Na]^+^. ^1^H NMR (400 MHz, pyridine-d_5_) *δ*_H_ 8.09 (d, *J* = 2.1 Hz, 1H, H-2′), 7.58 (d, *J* = 9.7 Hz, 1H, H-4), 6.69 (d, *J* = 2.1 Hz, 1H, H-3′), 6.53 (d, J = 9.7 Hz, 1H, H-3). ^13^C NMR (100 MHz, pyridine-d_5_) *δ*_C_ 161.2 (s, C-2), 148.3 (d, C-2′), 146.2 (s, C-7), 143.3 (d, C-4), 141.9 (s, C-5), 133.1 (s, C-8a), 127.7 (s, C-8), 116.6 (s, C-4a), 116.3 (s, C-6), 115.3 (d, C-3), 107.3 (d, C-3′).

Compound **11**: yellow solid. ESI-MS (positive ion) *m/z* 185 [M + Na]^+^. ^1^H NMR (400 MHz, CDCl_3_) *δ*_H_ 7.67 (d, *J* = 9.5 Hz, 1H, H-4), 7.42 (d, *J* = 8.0 Hz, 1H, H-5), 7.06 (m, 1H, H-6), 7.00 (m, 1H, H-8), 6.27 (d, *J* = 9.5 Hz, 1H, H-3). ^13^C NMR (100 MHz, CDCl_3_) *δ*_C_ 163.2 (s, C-7), 161.5 (s, C-2), 157.1 (s, C-9), 144.4 (d, C-4), 130.3 (d, C-5), 114.2 (d, C-6), 112.4 (d, C-3), 112.2 (s, C-10), 103.7 (d, C-8).

Compound **12**: white solid. ESI-MS (positive ion) *m/z* 215 [M + Na]^+^. ^1^H NMR (400 MHz, acetone-d_6_) *δ*_H_ 7.86 (d, *J* = 9.6 Hz, 1H, H-4), 7.26 (d, *J* = 8.4 Hz, 1H, H-5), 6.88 (d, J = 8.4 Hz, 1H, H-6), 6.18 (d, J = 9.6 Hz, 1H, H-3), 3.92 (s, 3H, OCH_3_). ^13^C NMR (100 MHz, acetone-d_6_) *δ*_C_ 160.9 (s, C-2), 154.5 (s, C-7), 149.2 (s, C-9), 145.3 (d, C-4), 135.4 (s, C-8), 124.5 (d, C-5), 113.7 (d, C-6), 113.0 (s, C-10), 112.7 (d, C-3), 61.5 (q, OCH_3_).

Compound **13**: yellow solid. ESI-MS (positive ion) m/z 215 [M + Na]^+^. ^1^H NMR (400 MHz, acetone-d_6_) *δ*_H_ 8.83 (s, 1H, OH), 7.84 (d, *J* = 9.5 Hz, 1H, H-4), 7.21 (s, 1H, H-5), 6.80 (s, 1H, H-8), 6.17 (d, J = 9.5 Hz, 1H, H-3), 3.90 (s, 3H, OCH_3_). ^13^C NMR (100 MHz, acetone-d_6_) *δ*_C_ 161.3 (s, C-2), 151.9 (s, C-8a), 151.2 (s, C-7), 146.1 (s, C-6), 144.7 (d, C-4), 113.3 (d, C-3), 112.2 (s, C-4a), 109.9 (d, C-5), 103.6 (d, C-8), 56.8 (q, OCH_3_).

Compound **14**: yellow solid. ESI-MS (positive ion) m/z 229 [M + Na]^+^. ^1^H-NMR (400 MHz, CD_3_OD) *δ*_H_: 7.86 (1H, d, *J* = 9.5 Hz, H-3), 7.12 (1H, s, H-5), 6.78 (1H, s, H-8), 6.20 (1H, d, *J* = 9.5 Hz, H-4), 3.88 (3H, s, 7-OCH_3_), 3.67 (3H, s, 6-OCH_3_); ^13^C-NMR (100 MHz, CD_3_OD) *δ*_C_: 162.2 (s, C-2), 153.1 (s, C-7), 151.7 (s, C-9), 146.5 (s, C-6), 143.7 (d, C-4), 112.6 (d, C-3), 112.2 (s, C-10), 109.2 (d, C-5), 102.4 (d, C-8), 56.1 (q, 7-OCH_3_), 53.1 (q, 6-OCH_3_).

### Antifungal assay

The inhibitory effects of compounds **1**–**14** on three fungi (*B. dothidea*, *F. oxysporum* and *P. oryzae*) were evaluated using a mycelial growth inhibition assay (Liu et al., 2009; Huang, et al., 2016). Briefly, dissolve the tested compound in acetone to form a compound solution with a concentration of 100ug/ml, and each compound solution was added to the PDA medium at approximate 50°C to give a culture dish with the toxic medium. After that, a 6-mm in diameter PDA disk with phytopathogen mycelium was transferred to the center of each culture dish, and the side with mycelia was downward. The dish containing an equal amount of acetone acted as solvent control. Each experiment was performed three times. All dishes were placed in a constant temperature incubator and cultured at 27°C. After 4 days, based on the cross method, the diameter of the pathogen growth circle was measured, and the mycelial growth inhibition rate was calculated according to the following formula:

$$Inhibition\left(\%\right)\frac{Average\;diameter\;of\;the\;control-Average\;diameter\;of\;the\;treatment}{Average\;diameter\;of\;the\;control}\times 100$$

## Results and Discussion

### Structure elucidation

Compound **1** was isolated as yellowish solid, its molecular formula was deduced to be C_25_H_24_N_2_O_5_ from its HR-ESIMS data (positive ions) (*m/z* 455.1584 [M + Na]^+^, calcd. 455.1582), indicative of 15 degrees of unsaturation. 1 gave IR absorption bands of -NH group at 3243 cm^−1^ and of an amide at 1641 cm^−1^. The ^1^H NMR spectrum of 1 (Table 1) contained signal at *δ*_H_ 6.08 (br s) assigned to the NH group, 7.54 and 6.98 (each, 1H, d, *J* = 2.3 Hz) assigned to H-24/H-23 on furan, 7.75 and 7.25 (each, 1H, d, *J* = 8.6 Hz) assigned to H-17/H-18 on benzene ring, 7.59 and 7.05 (each 1H, d, *J* = 8.3 Hz) assigned to H-12/H-11 on cis-olefin. Besides, the ^1^H NMR spectrum of 1 (Table 1) also revealed three methoxys (*δ*_H_ 4.35, 3H, s; 3.98, 3H, s and 3.69, 3H, s), two methylenes (*δ*_H_ 3.58, 2H, dd, *J* = 13.0, 6.5 Hz and *δ*_H_ 2.77, 2H, t, *J* = 6.5 Hz) and four aromatic protons of one disubstituted benzene ring (*δ*_H_ 7.38, 1H, t, *J* = 8.1 Hz; *δ*_H_ 7.34, 1H, overlapped; *δ*_H_ 6.96, 1H, d, *J* = 7.7 Hz and *δ*_H_ 6.76, 1H, d, *J* = 8.1 Hz). The ^13^C NMR spectrum (Table 1) revealed resonances for 25 carbons, attributable to three methoxys (*δ*_C_, 55.3, 56.0 and 59.0), one amide carbonyl (*δ*_C_, 167.5), two methylenes (*δ*_C_, 41.3 and 34.8), ten *sp* ^2^ methines (*δ*_C_, 104.6, 107.8, 2 × 114.2, 123.5, 126.8, 128.6, 129.8, 131.4, 143,9), nine *sp* ^2^ carbons (*δ*_C_, 103.9, 119.7, 130.9, 134.7, 137.6, 154.6, 156.9, 158.4, 163.3). The HSQC spectrum supported this assignment and allowed the association of all these carbons with the directly attached protons.

**FIGURE 2:**
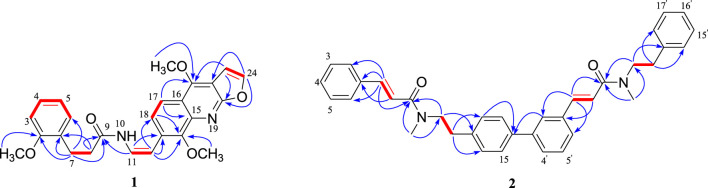
Key ^1^H–^1^H COSY and HMBC correlations of compounds **1** and **2**.

According to the correlations of the ^1^H–^1^H COSY spectrum, the ^1^H NMR multiplets could be classified into five spin systems (the red part in Figure 2). In order to establish the planar structure of 1, the HMBC correlations (Figure 2) were used to connect these fragments and locate quaternary carbons.

Specifically, the 3-phenylpropionyl was deduced from the HMBC correlations of H-7 with the *sp* ^2^ methine (C-6) and the amide carbonyl (C-9), and H-8 with the *sp* ^2^ quaternary carbon (C-1). After careful analysis of the remaining ^1^H and ^13^C NMR signals of **1**, it was inferred that there was also a structural fragment similar to skimmianine (4,7,8-trimethoxyfuro [2,3-b]quinoline) (Liu et al., 1991) in compound **1** in addition to a pair of cis double bonds. Comparison of the NMR spectroscopic data of this fragment with those of the known skimmianine established that the fragment had a very similar structure to the latter, but with a double bond replacing 13-methoxy. To confirm the location of the double bond, HSQC and HMBC experiments were conducted, in the HMBC spectrum (Figure 2), the correlations of H-12 with C-14 and C-18, and H-11 with C-13 supported the connection of the double bond to C-13. Ulteriorly, the HMBC correlation (Figure 2) of H-11 with C-9 showed that the 3-(2-methoxyphenyl)propanamido and the fragment similar to skimmianina were connected by the double bond. In addition, the HMBC (Figure 2) correlations of H-7 and H-(2-OCH_3_) with C-2 revealed that one methoxy was attached to C-2. Thus, the structure of clauphenamide A (**1**), with the unit of N-2-(4,8-dimethoxyfuro [2,3-b]quinolin-7-yl)vinyl, was determined as shown in Figure 1.

Compound **2**, a yellowish plates, was found to have a molecular formula of C_36_H_36_N_2_O_2_, which was deduced from the HRESIMS data (positive ions, m/z 551.2676 [M + Na]^+^, calcd. 551.2675). The ^1^H NMR spectrum of **2** (Table 1) contained two pairs of trans-olefinic protons signals (*δ*_H_ 7.61, 6.76, each 1H, d, *J* = 15.5 Hz and *δ*_H_ 7.45, 6.46, each 1H, d, *J* = 15.5 Hz), two pairs of methylenes coupled to each other (*δ*_H_ 3.60, 4H, d, *J* = 7.3 Hz and *δ*_H_ 2.82, 4H, t, *J* = 7.3 Hz) and two N-methyls (*δ*_H_ 2.95, 6H, s) (Lin, 1989). Thus, **2** was assigned as a phenylpropionamide (lansiumamide C) (Lin, 1989) dimer. The HMBC correlations of the H-2′and C-7′/C-16 implied that C-1′ was connected to C-16. So, the structure of clauphenamide B (**2**), the first phenylpropionamide dimer, was proposed as shown in Figure 1.

By comparing the spectral data of the 12 known compounds with those reported in the literature, their structures were identified as (-)-clausenamide (**3**) (Yang, et al., 1988), neoclausenamide (**4**) (Yang, et al., 1988), syringaresinol (**5**) (Ouyang et al., 2007), radicol (**6**) (Zhao et al., 2008), imperatorin (**7**) (Dien et al., 2012), 8-hydroxyfurocoumarin (**8**) (Kumar et al., 1995), (E,E)-8-(7-hydroxy-3,7-dimethylocta-2,5-dienyloxy)psoralen (**9**) (Ito et al., 1998), 5,8-dihydroxypsoralen (**10**) (Marumoto and Miyazawa, 2012), umbelliferone (**11**) (Baba et al., 1987), 7-hydroxy-8-methoxycoumarin (**12**) (Alexander et al., 1987), scopoletin (**13**) (Liu et al., 2011), scoparone (**14**) (Chen et al., 2010).

### Antifungal activity

The antifungal activities of compounds **1**–**14** were evaluated (Table 2) by a mycelial growth inhibition assay. At the concentration of 100 *μ*g/ml, compared with the control (chlorothalonil, inhibition rate of 83.67%), compounds **1** and **2** were found to exhibit moderate activity against *B. dothidea* with inhibition rate values of 68.39% and 52.05%, respectively. Compounds **11**–**14** showed antifungal activities to varying degrees against *B. dothidea*, *F. oxysporum* and *P. oryzae*, with inhibition rates greater than 40%. In addition, compared with the control (chlorothalonil, inhibition rate of 69.02%), compounds **11**–**14** showed strong antifungal activity to *P. oryzae*, with inhibition rates greater than 55%. Among them, compound **14** has the strongest antifungal activity against *P. oryzae*, and the inhibition rate (65.44%) is close to that of the control chlorothalonil. Compounds **5**–**10** showed weak antifungal activity against three kinds of fungi, and the inhibition rate was less than 15%.

**TABLE 2:** The antifungal activities of compound **1**–**14** against three fungi (100 μg/mL).

Compounds/fungi	Inhibition rate (%)±S.D.		
	*B. dothidea*	*F. oxysporum*	*P. oryzae*
**1**	68.37 ± 1.97 b	24.12 ± 0.88 e	28.38 ± 0.78 e
**2**	52.05 ± 2.02 cd	21.56 ± 0.97 f	25.07 ± 1.03 f
**3**	7.23 ± 0.98 h	6.21 ± 0.87 h	6*.*69 ± 0.85 i
**4**	8.45 ± 1.07 h	7.13 ± 0.79 h	6.24 ± 0.84 i
**5**	—	—	—
**6**	—	—	—
**7**	5.89 ± 1.01 h	—	—
**8**	11.31 ± 1.21 g	9.23 ± 1.02 g	8.92 ± 0.76 h
**9**	—	—	—
**10**	15.33 ± 0.89 f	10.74 ± 0.69 g	12.15 ± 0.89 g
**11**	45.76 ± 1.11 e	40.33 ± 1.09 d	55.67 ± 1.07 d
**12**	48.29 ± 0.98 de	43.74 ± 0.93 c	60.91 ± 1.10 c
**13**	49.88 ± 1.02 d	45.24 ± 1.04 c	62.08 ± 0.87 c
**14**	53.13 ± 1.08 c	48.39 ± 1.22 b	65.44 ± 1.04 b
chlorothalonil	83.67 ± 0.96 a	68.91 ± 0.79 a	69.02 ± 1.03 a

“—”, Inhibition rate <5%. Chlorothalonil was used as a positive control. *t* test, letters *p* < 0.05.

### Structure-activity relationship

**FIGURE 3:**
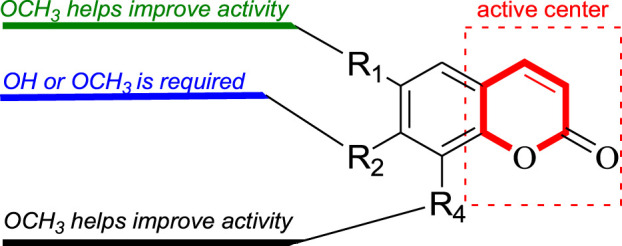
The structure-activity relationship analysis of simple coumarins.

In this study, Fourteen different types of compounds showed antifungal activities to varying degrees against *B. dothidea*, *F. oxysporum* and *P. oryzae*. Comparison of coumarins (**7**–**14**) suggests that the inhibitory activities of simple coumarins on three fungi are generally higher than that of furacoumarins. Further comparison of simple coumarins (**11**–**14**) reveals that the substitutions at positions 7 and 6 seem to be helpful to improve the antifungal activities of coumarins against three fungi, and methoxylations at positions 7 and 6 have better inhibitory effect on three fungi than hydroxylation. The structure-activity relationship of simple coumarins could be drawn as shown in Figure 3.

## Conclusion

In our present research, fourteen compounds were isolated from the twigs and leaves of *C. lansium*, among which compounds **1** and **2** were two novel amides. Compounds **5**, **6**, **10** and **12** were separated from the genus (*Clausena*) for the first time, while **13** was isolated in the species (*C. lansium*) for the first time. All isolated compounds were evaluated for their antifungal activities against *B. dothidea*, *F. oxysporum* and *P. oryzae*. As a result, clauphenamide A (**1**) and clauphenamide B (**2**) displayed moderate activity against *B. dothidea*. Umbelliferone (**11**), 7-hydroxy-8-methoxycoumarin (**12**), scopoletin (**13**), and scoparone (**14**) also exhibited moderate activity against *B. dothidea* and *F. oxysporum*. In addition, **11**–**14** showed strong antifungal activity against *P. oryzae*, among them, **14** has the strongest antifungal effect against *P. oryzae* and its inhibition rate is close to that of the control (chlorothalonil). Preliminary structure-activity relationship analysis revealed that: (1) Simple coumarins generally have higher antifungal effects than furacoumarins; (2) Methoxylations at positions 7 and 6 of simple coumarins have better inhibitory effects on fungi than hydroxylation.

## Data Availability

The original contributions presented in the study are included in the article/Supplementary Material, further inquiries can be directed to the corresponding author.
