# Supplementary material for: Antifungal active ingredient from the twigs and leaves of Clausena lansium Lour. Skeels (Rutaceae)
Source: Front Chem. 2022 Dec 13;10:1104805. doi: 10.3389/fchem.2022.1104805 (PMC9792782; doi:10.3389/fchem.2022.1104805)
Supplement: Supplementary file 1 [file DataSheet1.pdf]

Supporting Information

Antifungal Active Ingredient From the Twigs and Leaves of *Clausena lansium* Lour. Skeels (Rutaceae)

Xiaoxiang Fu [1#], Suling Xiao [1#], Duantao Cao [1], Minxuan Yuan [1], Qinghong Zhou [2], Yingjin Huang [2,3], Hongyi Wei [1] and Wenwen Peng [1,2]*

[1] *The Laboratory for Phytochemistry and Botanical Pesticides, College of Agriculture, Jiangxi Agricultural University, Nanchang 330045, China;*

[2] *Jiangxi Province Key Laboratory of Tuberous Plant Biology, Jiangxi Agricultural University, Nanchang 330045, China;*

[3] *Key Laboratory of Crop Physiology, Ecology and Genetic Breeding, Ministry of Education/Jiangxi Province, Jiangxi Agricultural University, Nanchang 330045, China*

** Correspondence to Wenwen Peng, The Laboratory for Phytochemistry and Plant-derived Pesticides, College of Agriculture, Jiangxi Agri-cultural University, Nanchang 330045, China; E-mail: wwpeng@jxau.edu.cn.*

[#] *Contributed equally to this study*

Contents of Supporting Information

Figure S1. ^{1}H NMR for compound **1** in $CDCl_3$.

Figure S2. ^{13}C NMR for compound **1** in $CDCl_3$.

Figure S3. HSQC for compound **1** in $CDCl_3$.

Figure S4. ^{1}H-^{1}H COSY for compound **1** in $CDCl_3$.

Figure S5. HMBC for compound **1** in $CDCl_3$.

Figure S6. HRESIMS for compound **1**.

Figure S7. ^{1}H NMR for compound **2** in $CDCl_3$.

Figure S8. ^{13}C NMR for compound **2** in $CDCl_3$.

Figure S9. HSQC for compound **2** in $CDCl_3$.

Figure S10. ^{1}H-^{1}H COSY for compound **2** in $CDCl_3$.

Figure S11. HMBC for compound **2** in $CDCl_3$.

Figure S12. HRESIMS for compound **2**.

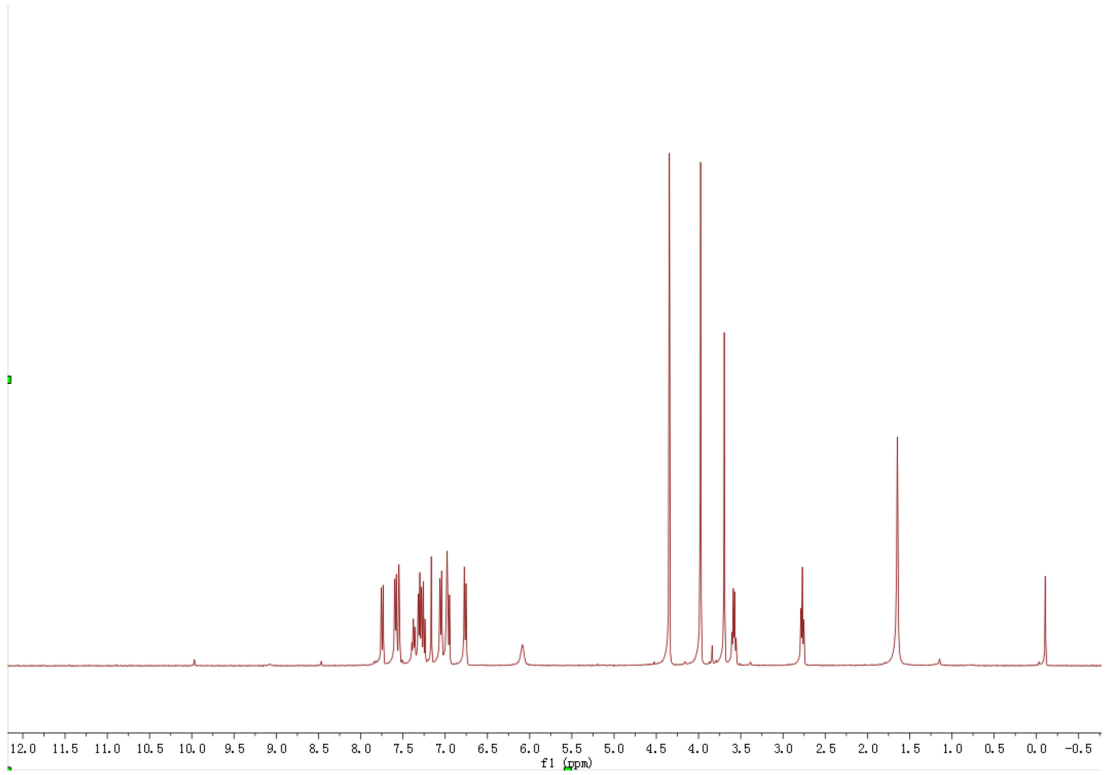

Figure S1. ^{1}H NMR for compound **1** in $CDCl_3$.

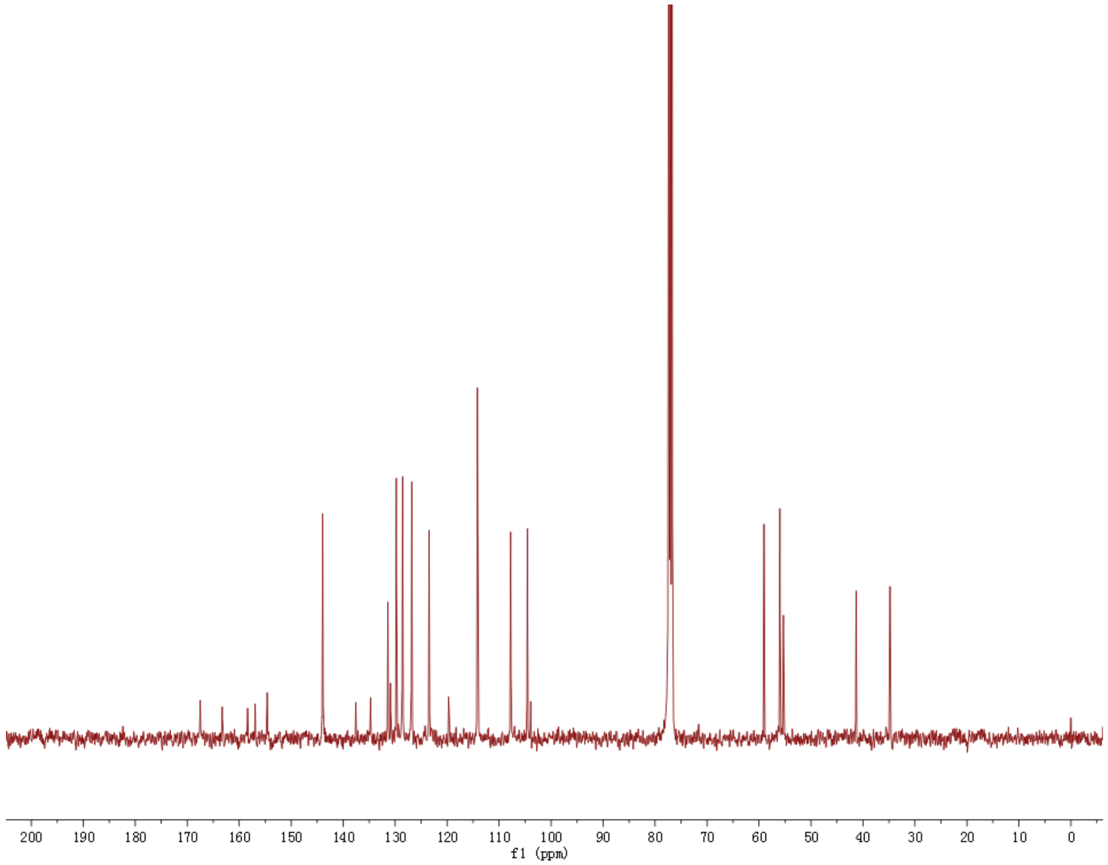

Figure S2. ^{13}C NMR for compound **1** in $CDCl_3$.

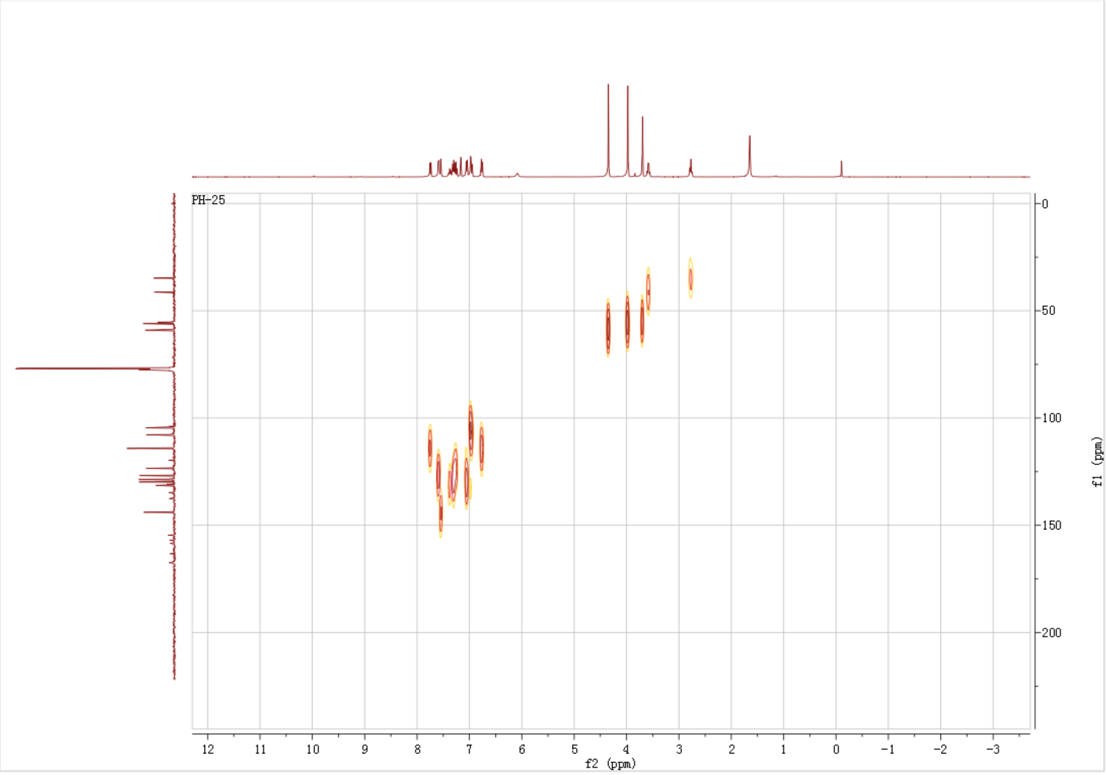

Figure S3. HSQC for compound **1** in $CDCl_3$.

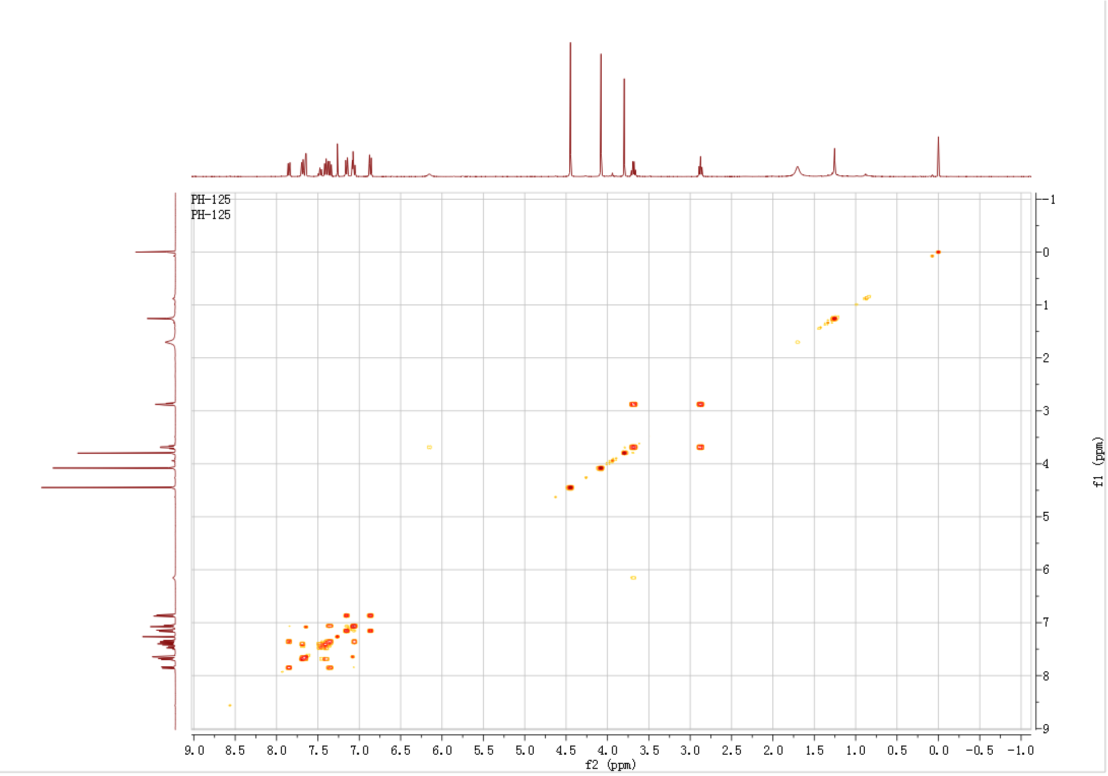

Figure S4. ^1H-^1H COSY for compound **1** in $CDCl_3$.

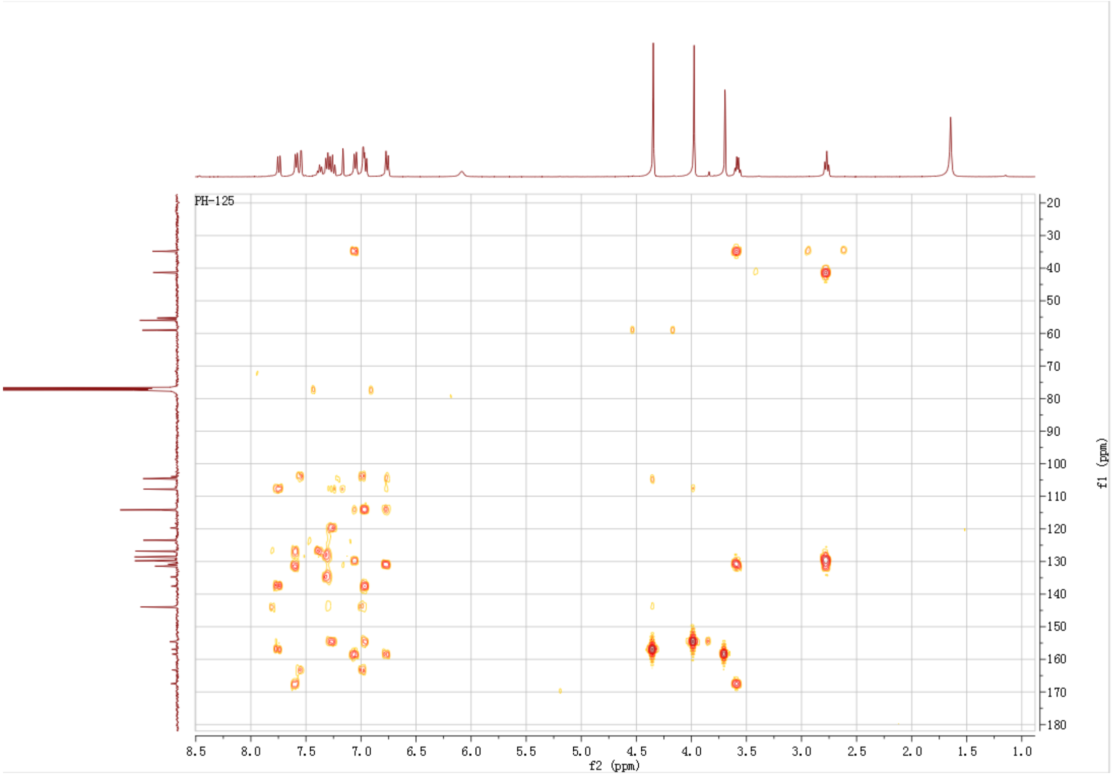

Figure S5. HMBC for compound **1** in $CDCl_3$.

Qualitative Analysis Report

**Data File**	PH125.d	**Sample Name**	PH125
**Sample Type**	Sample	**Position**	P1-F3
**Instrument Name**	Instrument 1	**User Name**	
**Acq Method**	1-pos-4min.m	**Acquired Time**	7/14/2022 2:18:27 PM
**IRM Calibration Status**	Success	**DA Method**	11.m
**Comment**			
**Sample Group**		**Info.**	
**Stream Name**	LC 1	**Acquisition SW Version**	6200 series TOF/6500 series Q-TOF B.06.01 (B6172 SP1)

User Chromatograms

**Fragmentor Voltage**	175	**Collision Energy**	0	**Ionization Mode**	ESI

**Fragmentor Voltage**	**Collision Energy**	**Ionization Mode**
175	0	ESI

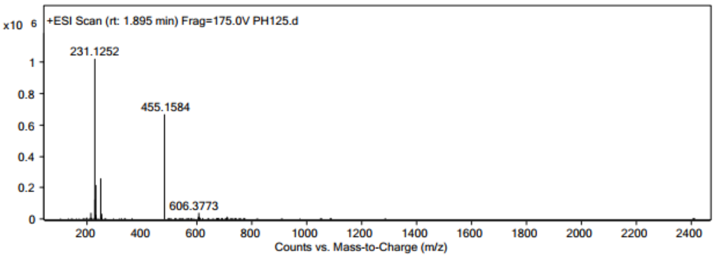

Formula (M)	Ion Formula	Mass (MFG)	m/z (Calc)	err (ppm)
C25H24N2O5	C25H324N2O5	455.1584	455.1582	-0.4

Figure S6. HRESIMS for compound **1**.

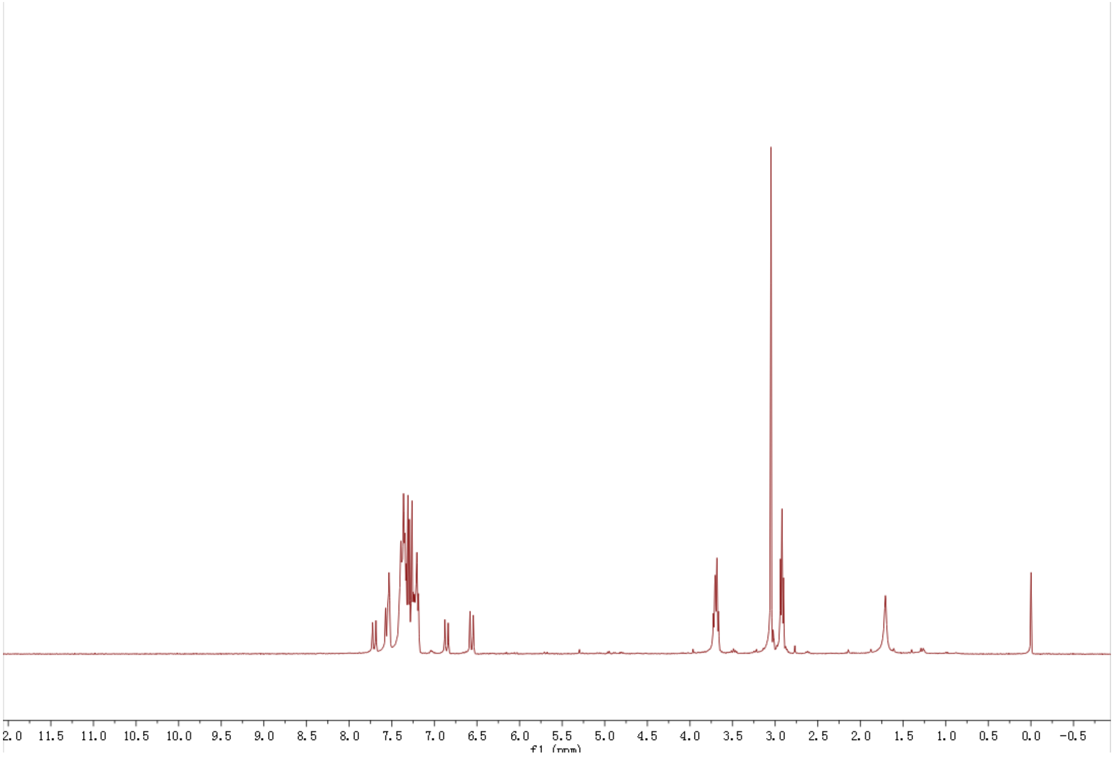

Figure S7. ^{1}H NMR for compound **2** in $CDCl_3$.

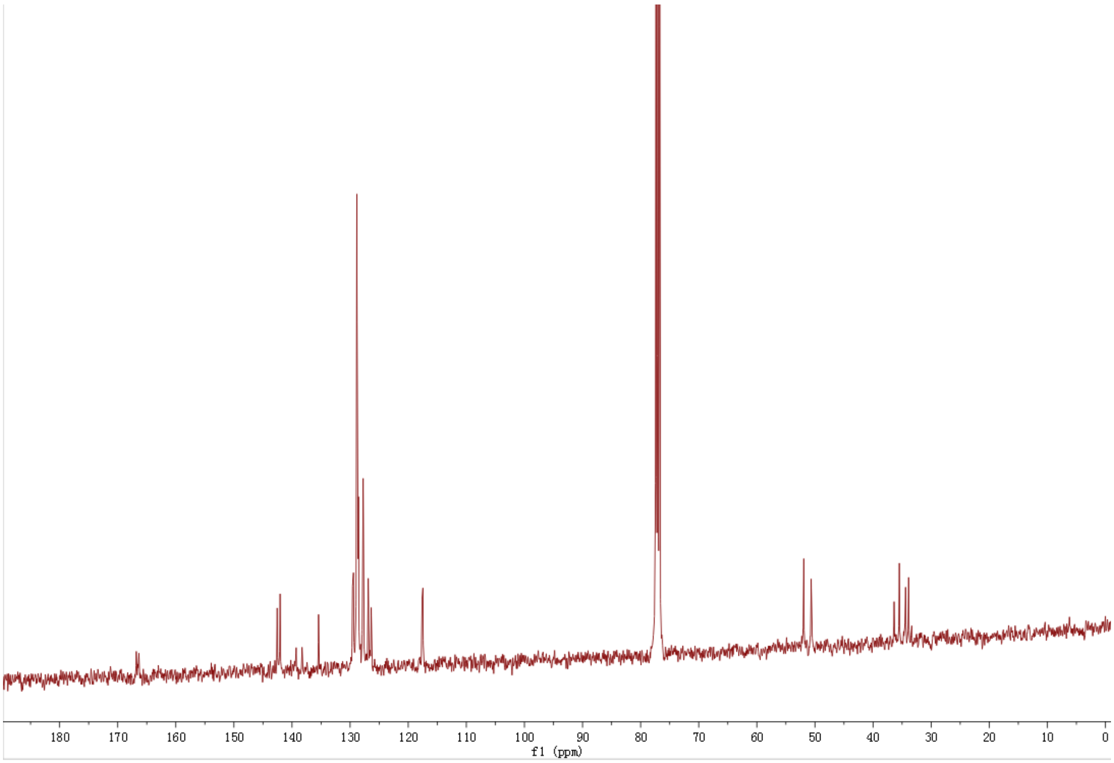

Figure S8. ^{13}C NMR for compound **2** in $CDCl_3$.

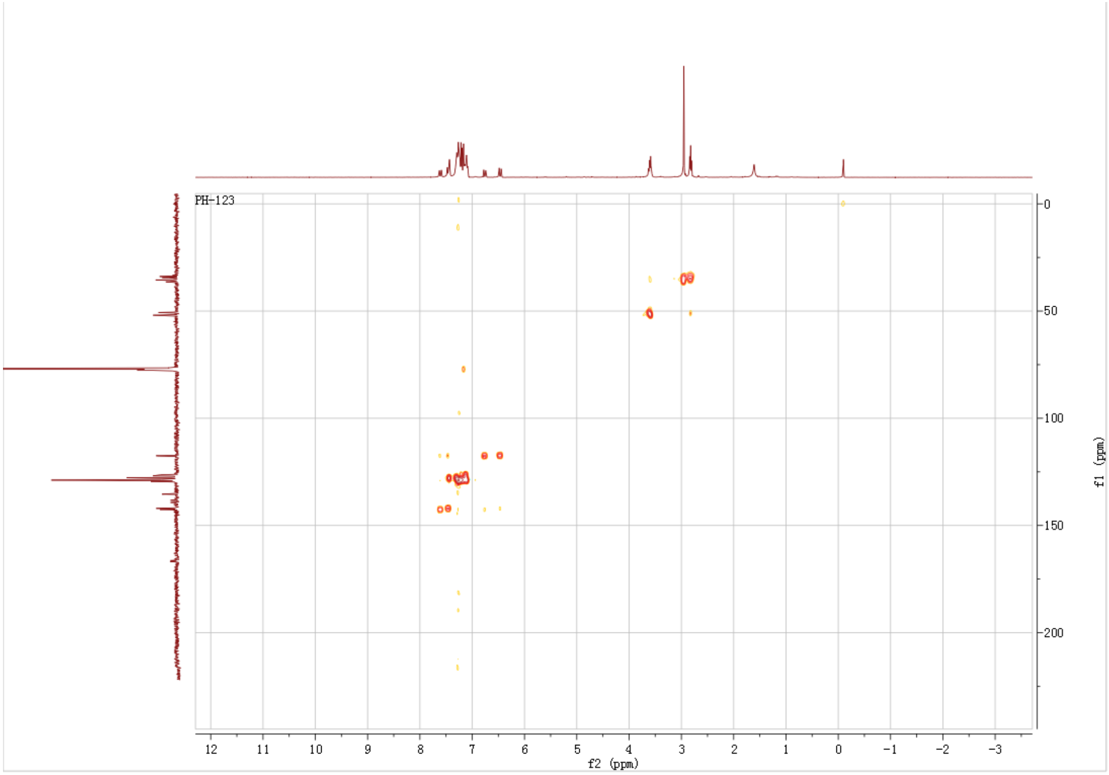

Figure S9. HSQC for compound **2** in CDCl3.

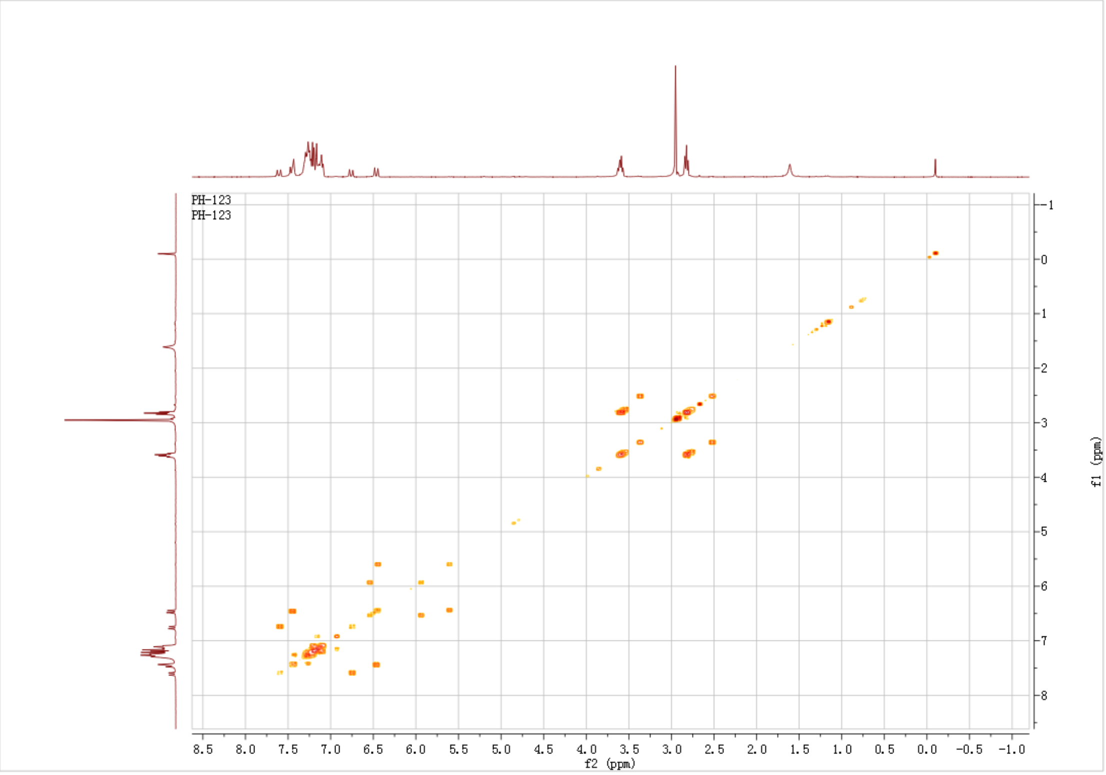

Figure S10. ^{1}H-^{1}H COSY for compound **2** in CDCl3.

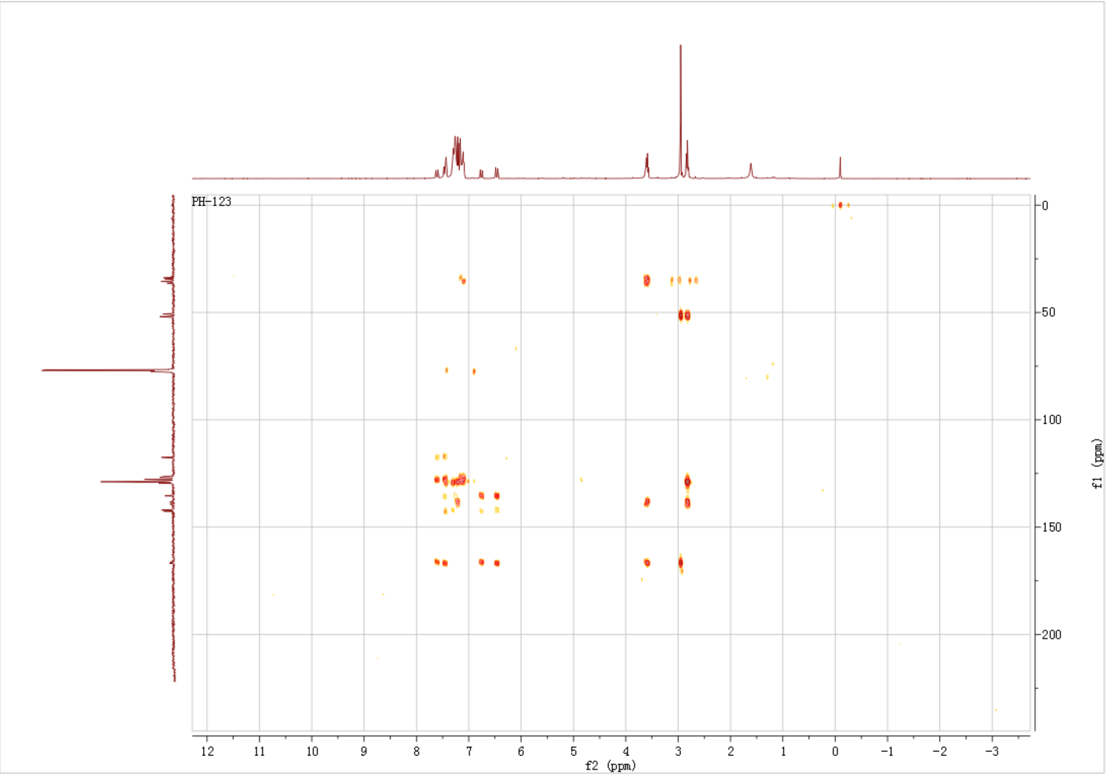

Figure S11. HMBC for compound **2** in $CDCl_3$.

Qualitative Analysis Report

**Data File**	PH123.d	**Sample Name**	PH123
**Sample Type**	Sample	**Position**	P1-F2
**Instrument Name**	Instrument 1	**User Name**	
**Acq Method**	1-pos-4min.m	**Acquired Time**	7/14/2022 2:10:41 PM
**IRM Calibration Status**	Success	**DA Method**	11.m
**Comment**			
**Sample Group**		**Info.**	
**Stream Name**	LC 1	**Acquisition SW Version**	6200 series TOF/6500 series Q-TOF B.06.01 (B6172 SP1)

User Chromatograms

Fragmentor Voltage 175 **Collision Energy** 0 **Ionization Mode** ESI

Fragmentor Voltage	Collision Energy	Ionization Mode
175	0	ESI

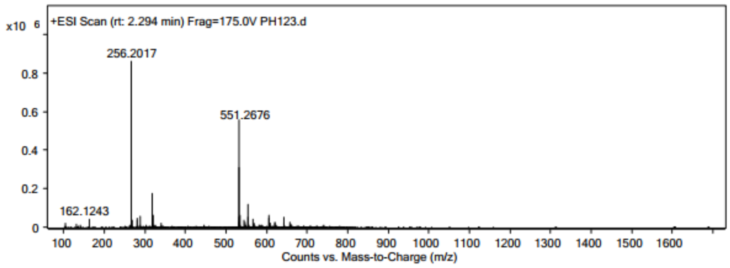

Formula (M)	Ion Formula	Mass (MFG)	m/z (Calc)	err (ppm)
C20H25	C20H26	551.2676	551.2675	0.2

Figure S12. HRESIMS for compound **2**.
